# Effect of phosphorus deficiency on biomass and root system architecture in diverse *Medicago* accessions

**DOI:** 10.3389/fpls.2026.1812278

**Published:** 2026-04-23

**Authors:** Nagarjun Devabhakthini, Bettina Eichler-Löbermann, Reza Haghi, Evelin Willner, Klaus J. Dehmer, Mareike Kavka

**Affiliations:** 1 Genebank Department, Satellite Collections North, Leibniz Institute of Plant Genetics and Crop Plant Research (IPK), Malchow, Germany; 2 Chair of Agronomy and Crop Science, University of Rostock, Rostock, Germany; 3 Genebank Department, Domestication Genomics, Leibniz Institute of Plant Genetics and Crop Plant Research (IPK), Gatersleben, Germany

**Keywords:** alfalfa, k-mer GWAS, phosphorus efficiency, plant genetic resources, rhizotron

## Abstract

**Introduction:**

Phosphorus (P) is essential for legume growth and symbiotic nitrogen fixation, yet its low availability in many soils frequently constrains plant productivity.

**Methods:**

To evaluate phenotypic and genotypic variation in P efficiency, 200 genetically diverse accessions of the *Medicago sativa* complex were assessed under high and low P conditions in a greenhouse experiment, followed by detailed root system architecture (RSA) analysis in a subset of 20 accessions using a rhizotron system.

**Results:**

Significant intraspecific variation was observed for shoot and root dry matter, root-to-shoot ratio, shoot P content, and P utilisation efficiency (PUE). Across the full panel of 200 accessions, shoot and root dry matter decreased by 24% and 23% respectively under low P, and in the subset of 20 accessions PUE increased by 38% under low P. Genome-wide association studies identified candidate genes associated with plant height and biomass related traits under high and low P conditions including genes involved in cell-wall modification, hormonal regulation, and growth-related stress responses. Rhizotron analyses revealed RSA plasticity, with increased specific root length and specific convex hull area under low P, reflecting morphological adjustments that enhance soil exploration.

**Discussion:**

Accessions that combined stable biomass production, high PUE, and adaptive RSA features under low P represent valuable genetic resources for breeding nutrient-efficient alfalfa cultivars suitable for low-input agricultural systems.

## 1 Introduction

Alfalfa (*Medicago sativa* L.) is a widely cultivated perennial forage legume, valued for its high biomass yield, rich protein concentration, superior forage quality, and beneficial ecological contributions to sustainable agricultural systems (Annicchiarico, 2015; Julier et al., 2015; Zhang and Wang, 2025). Alfalfa is a member of the *M. sativa* complex, which includes taxa closely related to alfalfa with overlapping morphological traits and extensive interspecific hybridisation (Quiros and Bauchan, 1988; Şakiroğlu and İlhan, 2021). It has a small seed size, relatively slow seedling establishment and extensively relies on biological nitrogen (N) fixation, particularly under low external N input conditions. Through symbiotic association with *Rhizobium* spp., alfalfa can fix atmospheric N at rates exceeding 300 kg N ha^-^¹ year^-^¹, reducing reliance on synthetic N fertilisers and enhancing soil fertility (Carlsson and Huss-Danell, 2003; Barbieri et al., 2023).

However, alfalfa growth and N fixation remain highly sensitive to abiotic stresses particularly phosphorus (P) limitation, which constrains several energy-intensive processes required for plant development (Fan et al., 2016). Phosphorus availability strongly influences the efficiency of biological N fixation, as the nitrogenase enzyme responsible for reducing N_2_ to ammonia requires at least 16 ATP molecules per reaction (Hubbell and Kidder, 2003; Vance et al., 2003). Inadequate P supply restricts root and nodule development, impairs carbon allocation, and reduces overall N-fixation efficiency and productivity in legumes, including alfalfa (Vance et al., 2003). Due to its high biomass production and efficient symbiotic N fixation, *M. sativa* has a comparatively high P demand and is therefore sensitive to P deficient soil conditions (Vance et al., 2003; Yang et al., 2014; Hu et al., 2024).

Globally, inefficient P fertiliser use poses significant agronomic and environmental challenges. Less than 50% of applied P is typically absorbed directly by crops within the application year, with the remainder becoming less available as organic or inorganic complexes or lost through runoff, contributing to eutrophication in aquatic ecosystems (Shen et al., 2011; Walsh et al., 2023). Additionally, phosphate rock which is the primary source of mineral P fertilisers is a finite resource, highlighting the urgency to enhance P acquisition efficiency (PAE) and P utilisation efficiency (PUE) in crops (Horst et al., 2001; Pang et al., 2018). Here, PAE reflects the plant’s ability to acquire P from the soil, whereas PUE indicates how efficiently the absorbed P is utilised for biomass production (Wang et al., 2010; Chen et al., 2023).

Under P limited conditions, plants adopt morphological and physiological strategies to optimise P uptake. These include modifications of root system architecture (RSA), root exudation of organic acids and phosphatases, and increased symbiotic associations with arbuscular mycorrhizal fungi (Lynch, 2011; Richardson et al., 2011; Zhang et al., 2024). Typically, alfalfa develops a dominant taproot system, which may be less optimal for accessing P rich upper soil layers. However, adaptive root modifications such as increased lateral root formation, greater total root length and higher root-to-shoot ratios (RSR) have been documented as effective strategies for improved P acquisition (Fan et al., 2015; Pan et al., 2023; Fan et al., 2025). Considerable variability in RSA responses to P availability has been found in several crop species such as sorghum (Parra-Londono et al., 2018), potato (Kirchgesser et al., 2023), and alfalfa (Bucciarelli et al., 2021; Hu et al., 2023; Pan et al., 2023), indicating potential targets for breeding P efficient cultivars.

Nevertheless, current knowledge of P efficiency in the *M. sativa* complex remains fragmented. Studies on individual cultivars or a small number of genotypes have shown that low P, alone or in combination with drought, induces changes in root morphology and physiology, including shifts in specific root length, biomass allocation and carboxylate exudation, with consequences for P uptake and PUE (Fan et al., 2015). Separately, rhizotron and seedling phenotyping studies, as well as root-crown imaging of field-grown plants, have shown substantial genotypic variation and moderate-to-high heritability for RSA traits in alfalfa (Bucciarelli et al., 2021; Pan et al., 2023). For an objective classification of root types in alfalfa, the deployment of image-based and machine-learning approaches have recently been introduced (Xu et al., 2022). However, these efforts typically involve relatively small panels and typically focus on RSA of seedlings, and do not integrate biomass production, shoot P accumulation and PUE across a genetically diverse germplasm set under decreased P supply.

At the same time, genome-wide association studies (GWAS) in alfalfa have successfully identified the genetic basis of variation in agronomic performance, forage quality and plant height under field and controlled conditions (Biazzi et al., 2017; Jiang et al., 2024; Medina et al., 2025). Yet, conventional SNP-based GWAS remains constrained by its dependence on a single reference genome, which limits the detection of structural variants and present or absent polymorphisms especially relevant in an autotetraploid, outcrossing species with high genomic complexity such as in alfalfa (Cortes et al., 2021). Alignment-free, k-mer-based GWAS provides a complementary alternative by directly associating short sequence fragments with phenotypic variation and thereby capturing a broader spectrum of genomic polymorphisms, including those absent from current reference assemblies (Voichek and Weigel, 2020). This is especially relevant for alfalfa, where high heterozygosity, polyploidy, and structural variation can result in incomplete representation of genetic diversity in a single reference genome and complicate SNP-based approaches that rely on alignment and variant calling. Reference-independent approaches like k-mer-based GWAS can therefore capture a broader spectrum of genetic variation, including structural variants and sequences absent from the reference, making them particularly suitable for dissecting complex traits in this species (Long et al., 2022). Despite this potential, such approaches have not yet been applied to investigate plant height, biomass-related traits, or stress tolerance indices under contrasting P conditions in *M. sativa* germplasm.

This study aims to investigate the phenotypic and genotypic variation related to P efficiency in a set of *Medicago* accessions. Specifically, the objectives are: (1) to evaluate early plant growth and biomass production in response to low P conditions in 200 diverse *Medicago* accessions; (2) to identify genomic regions associated with plant growth and biomass related traits through k-mer GWAS in control and low P conditions; and (3) to assess P uptake and RSA responses in selected accessions. By characterising the accessions based on P responsive growth traits, this study provides a foundation for the development of alfalfa cultivars with improved performance under P deficient conditions.

## 2 Materials and methods

### 2.1 Plant material

The *Medicago* germplasm collection at the German Federal *Ex situ* Genebank of the Leibniz Institute of Plant Genetics and Crop Plant Research (IPK) comprises 1,234 accessions representing 40 species with diverse geographic origins and biological statuses (Devabhakthini et al., 2026). The present study focused on the *M. sativa* complex, which is of particular agronomic importance but taxonomically challenging. This complex includes the taxa *M. polychroa* Grossh., *M. hemicycla* Grossh., and *M. glutinosa* as well as *M. sativa* subsp. *sativa*, *M. sativa* subsp. *caerulea*, *M. sativa* nothosubsp. *varia* and *M. falcata* L., which most experts now consider as a single species with subspecies (Quiros and Bauchan, 1988; Şakiroğlu and İlhan, 2021). In this study, the nomenclature follows the IPK Genebank Information System (GBIS, https://gbis.ipk-gatersleben.de/gbis2i/), recognising *M. sativa*, *M. falcata*, and *M. × varia* as distinct species, to maintain consistency with genebank records.

To ensure genetic diversity and minimise redundancy, accession selection was guided by genotyping-by-sequencing (GBS) data (as described in Devabhakthini et al., 2026). Genetic distance analysis was performed on the 762 accessions in the IPK genebank from the *M. sativa* complex using an unlinked SNP dataset generated via the iPyrad pipeline (Eaton and Overcast, 2020). The dataset was converted to NEXUS format and analysed in PAUP* (Swofford, 2002), where pairwise genetic distances were calculated using uncorrected p-distances. To reduce redundancy, accessions exhibiting high sequence similarity (≥ 99.97%) were identified using the mergeTaxa function, with the accession containing the least missing data retained from each redundant pair. To capture a broad and representative sampling of genetic diversity within the *M. sativa* complex, three iterative rounds of filtering and selection were performed. This procedure resulted in the removal of 558 redundant accessions, yielding a final panel of 203 genetically distinct accessions. From this panel, a subset of 200 accessions was selected for phenotypic evaluation under contrasting P conditions. These comprised 76 *M. sativa* subsp. *sativa*, seven *M. sativa* subsp. *caerulea*, 41 *M. sativa* (subspecies not determined), 69 *M.* × *varia*, two *M. falcata*, two *M. glutinosa*, two *M. polychroa*, and one *M. hemicycla* accession. The selected accessions originated from diverse geographical regions and included cultivars, landraces, breeding materials, and wild populations, based on passport data retrieved from the GBIS on February 11, 2026 (**Supplementary Table 1**).

### 2.2 Greenhouse experiment for phenotypic characterisation of PAE and PUE

A greenhouse experiment was conducted to assess variation in biomass production among the 200 genetically diverse accessions of the *M. sativa* complex under two P supply conditions: high P (HP) and low P (LP). Seeds were germinated under controlled conditions for 10 days in a GS10/11 germination chamber (Flohr Instruments, Nieuwegein, The Netherlands), with an 11-hour light period at 22 °C and a 13-hour dark period at 15 °C. Eleven-day-old seedlings were transplanted into 7 L pots at an initial density of 13 plants per pot, which was thinned to 10 plants per pot after one week.

The growth substrate consisted of a mixture of 60% commercial potting soil and 40% perlite (Klasmann-Deilmann GmbH, Geeste, Germany). The substrate was supplemented with essential macronutrients except P by the manufacturer. The final mix had a pH of 6.0–6.5 (measured in H_2_O). Before planting, P was supplied as triple superphosphate (TSP) placed 10–15 cm below the surface: 1 g total P per pot (5 g TSP) for HP and 0.05 g total P per pot (0.25 g TSP) for LP. Each accession was grown under both P conditions arranged such that HP and LP treatments were placed side by side to account for positional effects. Three replicates were planted in 2023 in consecutive batches: replicate 1 on April 20, replicate 2 on May 11, and replicate 3 on June 1, and all were grown for 46 days before shoot harvest. Shoots were harvested on a single day for each replicate. Root harvesting started immediately after shoot harvest and was completed over the following two days due to the time required for careful root extraction and washing for all replicates. Plants were irrigated regularly throughout the growth period to maintain optimal conditions. Directly before harvesting, plant height (minimum and maximum per pot) was recorded. Shoots and roots, pooled per pot, were harvested separately with shoot fresh weight recorded immediately. Roots were carefully extracted by inverting pots and manually shaking off excess substrate, thoroughly washed and dried using cloth towels to remove surface moisture. Shoots and roots were dried at 60 °C for 24 hours before recording dry weight.

### 2.3 k-mer based genome-wide association study

A k-mer GWAS was performed directly using raw genotyping-by-sequencing (GBS) reads. The used sequence data from our previous study (Devabhakthini et al., 2026) are available in the european nucleotide archive (ENA) under accession number PRJEB89658. For each sample, 31-bp k-mers were identified using KMC v3, a fast and memory-efficient k-mer counting tool for large-scale sequencing data (Kokot et al., 2017), and only canonical k-mers with a minimum count of five were retained. Strand information was assigned by comparing canonical and non-canonical k-mer counts across samples, and a combined presence–absence matrix was constructed. To ensure statistical reliability, k-mers with a minor allele frequency (MAF) < 0.01 were excluded. Population structure was accounted for by calculating a kinship matrix directly from the k-mer matrix. Association analyses were conducted for plant height, shoot fresh matter, shoot dry matter (SDM), root dry matter (RDM), and RSR under HP and LP conditions, as well as stress tolerance indices (STI) for shoot fresh matter, SDM and RDM using a linear mixed model implemented in GEMMA. The tested k-mer (presence/absence) was included as a fixed effect together with an intercept representing the overall mean phenotype, while a kinship matrix derived from the k-mer data was included as a random effect to account for population structure and relatedness among individuals. In the association analysis, k-mers were treated as binary markers (presence–absence), and associations were tested using a likelihood ratio test. A significance threshold of −log10(p-value) > 5 was used as the primary criterion for declaring significant associations. For traits where no k-mers exceeded this threshold, the top-ranking k-mers representing the strongest association signals (−log10(p-value) ≥ 4) were retained for downstream fragment extension and candidate gene identification. Significant k-mers were aligned to one another to extend k-mer fragments, as multiple significant k-mers may originate from the same sequencing reads and overlap in sequence. The resulting k-mer fragments were mapped to the *Medicago sativa* cv. *Zhongmu*-4 reference genome (Long et al., 2022) to identify genomic regions corresponding to linkage disequilibrium (LD) blocks, defined here as a 100 kb window. To characterise the biological relevance of the identified signals, functional annotations were compiled for all genes located within the associated LD blocks. Protein and genomic sequences were extracted from the reference genome and queried against the UniProt database using BLASTp and BLASTx to identify homologous proteins. Functional descriptions of the proteins were curated from multiple public databases to determine the biological roles. Candidate genes were prioritised based on their functional annotations and existing literature on the genetic background of the trait.

### 2.4 Detailed analyses on a subset of accessions

From the 200 accessions, root dry matter (RDM) difference was calculated as ΔRDM = mean RDM under HP − mean RDM under LP (means from three biological replicates per treatment). All accessions were ranked by ΔRDM. From the 30 accessions with largest differences and the 30 accessions with smallest differences, ten accessions were selected from each group to represent different biostatus categories (cultivar/landrace/wild) and geographic origins. These 20 contrasting accessions (**Supplementary Table 1**) were used for subsequent analyses of shoot P concentration, PUE and RSA. Root P concentration was not analysed because accurate determination is highly sensitive to residual substrate particles and to selective loss of fine roots during washing. Whereas this can substantially bias concentration-based measurements, such effects have limited impact on bulk traits like root dry matter. Moreover, shoot P concentration is commonly used to quantify plant P status and the outcome of P acquisition and translocation to aboveground biomass (Lyu et al., 2016). Dried shoot samples were milled into a fine powder using a Retsch MM 400 mixer mill (Retsch GmbH, Haan, Germany). The milled samples were placed in porcelain crucibles, dried at 105 °C, weighed, and then ashed at 550 °C for 4–5 hours in a muffle furnace. Total P was extracted using 25% hydrochloric acid according to Page et al. (1982), and P concentrations were measured via inductively coupled plasma-atomic emission spectroscopy (ICP-OES Optima 8300, Perkin Elmer, Waltham, MA, USA) at a wavelength of 214 nm and is given as mg P per gram dry weight after drying at 60 °C.

Root system architecture of the 20 selected accessions was investigated using rhizotrons measuring 43 cm (height) × 29 cm (width) × 2 cm (depth), with an approximate substrate volume of 2.55 liters (manufactured by Kunze Kunststoffe GmbH, Bentwisch, Germany). Each rhizotron featured a transparent front panel for root visualisation and drainage holes at the bottom to prevent waterlogging (**Supplementary Figure 1a**). The same substrate composition used in the pot experiment (60% potting soil and 40% perlite, v/v) was also used in the rhizotron experiment. Phosphorus was supplied at two levels: For the HP treatment, the P application rate used in the pot experiment was scaled to substrate volume in the rhizotrons, resulting in 0.37 g P per rhizotron (1.83 g TSP). For the LP treatment, the same absolute amount of P applied in the pot experiment (0.05 g P, equivalent to 0.25 g TSP) was applied per rhizotron to ensure an even distribution of TSP granules. Fertiliser granules were placed 1–2 cm below the substrate surface, and rhizotrons were positioned horizontally throughout the experiment. A nylon sheet was placed over the substrate before attaching the transparent front panel. Rhizotrons were arranged at an angle of about 40° with the front panel showing downwards, allowing one unit to shade the next. The final rhizotron in each box, which would otherwise be exposed to light, was covered with a cardboard sheet. Two ten-day-old seedlings per rhizotron were gently positioned between the nylon sheet and the transparent plate (**Supplementary Figure 1b**). Each accession was subjected to both P treatments, with five replicates per treatment. Replicates were arranged in completely randomised blocks, with blocks planted and harvested on consecutive days to manage planting and harvesting. Seedlings were grown for three weeks under greenhouse conditions, planted from October 23 to 27, 2023, and harvested between November 20 and 24, 2023. To ensure uniform soil moisture, rhizotrons were watered at regular intervals using wide-neck syringe bottles.

At the end of the experimental period, shoots were harvested, and fresh weight was immediately recorded. Root systems were carefully transferred to a flatbed scanner (Opticslim 1180, Plustek Inc., New Taipei City, Taiwan) and scanned at 600 dpi resolution to capture detailed structural information. Shoots and roots were dried at 60 °C, and weighed to determine dry weight. The scanned images were analysed using semi-automated Root Image Analysis (saRIA) software (Narisetti et al., 2019) to quantify key RSA parameters (**Supplementary Figure 1b**). Pixels were converted to mm using the scanning resolution.

### 2.5 Data analysis

Total phosphorus content in the shoot was calculated as:


$$\text{Shoot P content }({\text{mg pot}}^{-1})=\text{shoot dry matter }({\text{g pot}}^{-1})\;\times \text{ shoot P concentration }({\text{mg g}}^{-1})\;$$

Phosphorus utilisation efficiency (PUE) was calculated as the ratio of shoot dry matter (g) to the P content in the shoot (mg).


$$\text{Phosphorus utilisation efficiency }({\text{g DM mg}}^{-1\;}\text{P})\;=\frac{\text{shoot dry matter }({\text{g pot}}^{-1})}{\text{shoot P content }({\text{mg pot}}^{-1})}$$

For shoot fresh matter, shoot and root dry matter, stress tolerance indices (STI) were calculated according to Fernandez (1992):


$$\text{STI}=\frac{{\text{Y}}_{\text{pi }}\times {\text{Y}}_{\text{si}}}{{\text{Mean Y}}_{\text{p}}^{2}}$$

where Y_pi_ is the performance of the i^th^ population under control conditions (HP); Y_si_ is the performance of the i^th^ population under stress conditions (LP) and Mean Y_p_ is the mean performance of all the populations under control conditions (HP).

All phenotypic traits were analysed in R (version 4.2.2; R Core Team, 2022). Descriptive statistics, including mean and standard deviation, were calculated to characterise trait distributions and variability among accessions. Relationships between shoot and root STI values were assessed using Spearman correlation analysis. For selected analyses, a subset of 20 accessions representing the extremes of the root dry matter response (ΔRDM) to P limitation was defined and classified into “High ΔRDM” and “Low ΔRDM” groups. To evaluate the effects of P treatment, group and their interaction, analysis of variance (ANOVA) was performed using linear mixed-effects models implemented in the packages “lme4” (Bates et al., 2015) and “lmerTest” (Kuznetsova et al., 2017). In these models, P treatment, ΔRDM group, accession, and the treatment × group interaction was included as fixed effects, while replication was treated as a random effect to account for environmental variation. This mixed-model framework was applied to shoot P content and PUE. For rhizotron-derived RSA traits, linear mixed-effects models were fitted separately for each trait, including treatment, accession, and their interaction (treatment × accession) as fixed effects and replication as a random effect. To visualise multivariate relationships among RSA traits, all variables over both treatments were z-score scaled. A heatmap was generated using the heatmap.2 function (“gplots”; Warnes et al., 2022), which performs hierarchical clustering of both accessions and traits based on Euclidean distance and complete linkage, grouping accessions according to similarity in trait expression under HP and LP conditions.

## 3 Results

### 3.1 Plant performance under P deficiency in the pot experiment

**Figure 1:**
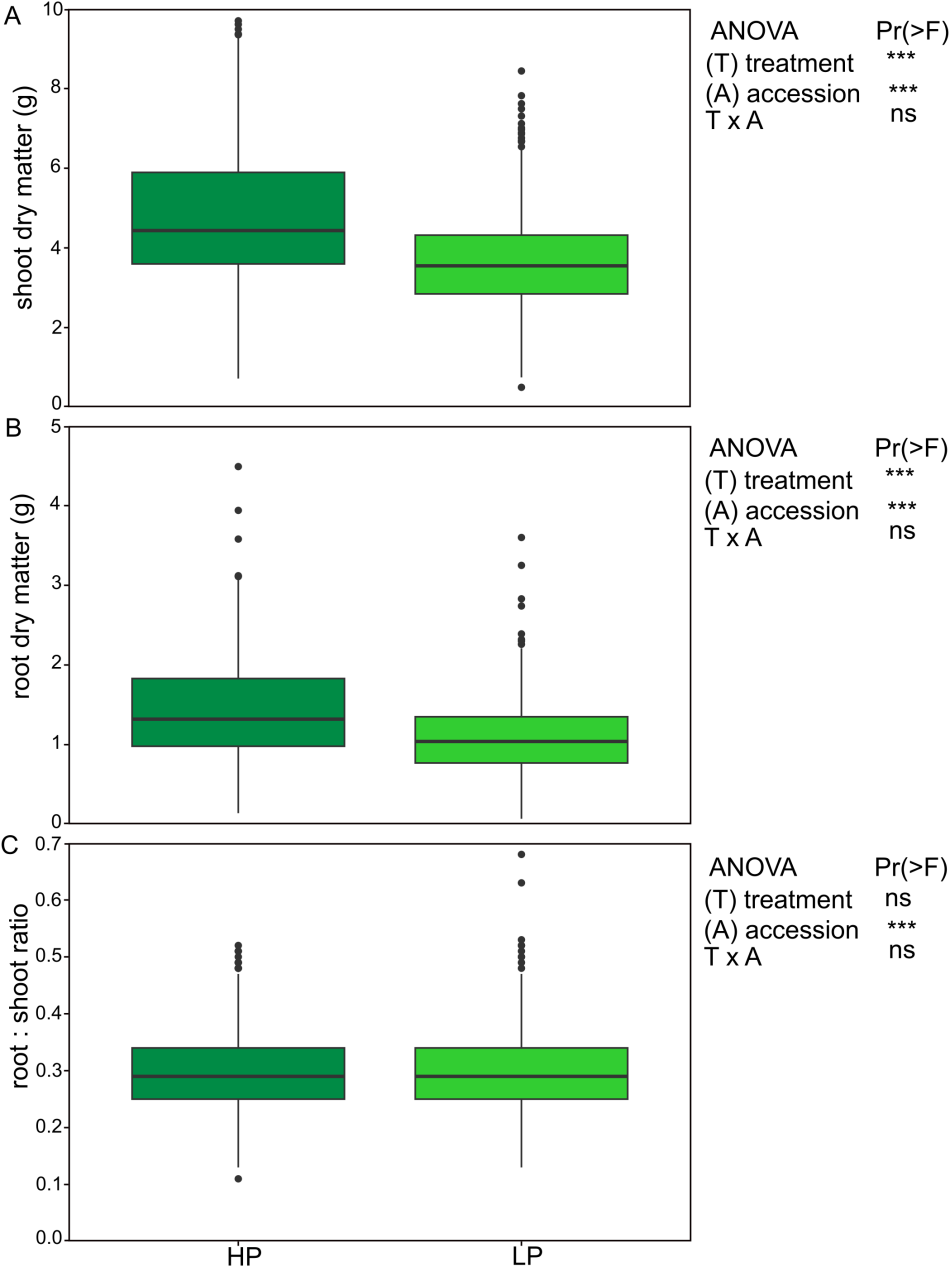
Boxplots showing the distribution of **(A)** shoot dry matter, **(B)** root dry matter, and **(C)** root-to-shoot ratio across two P treatments for 200 *Medicago* accessions (per pot; average of 3 replicates). Each box represents the interquartile range (IQR), with the median shown as a horizontal line. *** denote a significance value of p < 0.001.

**Figure 2:**
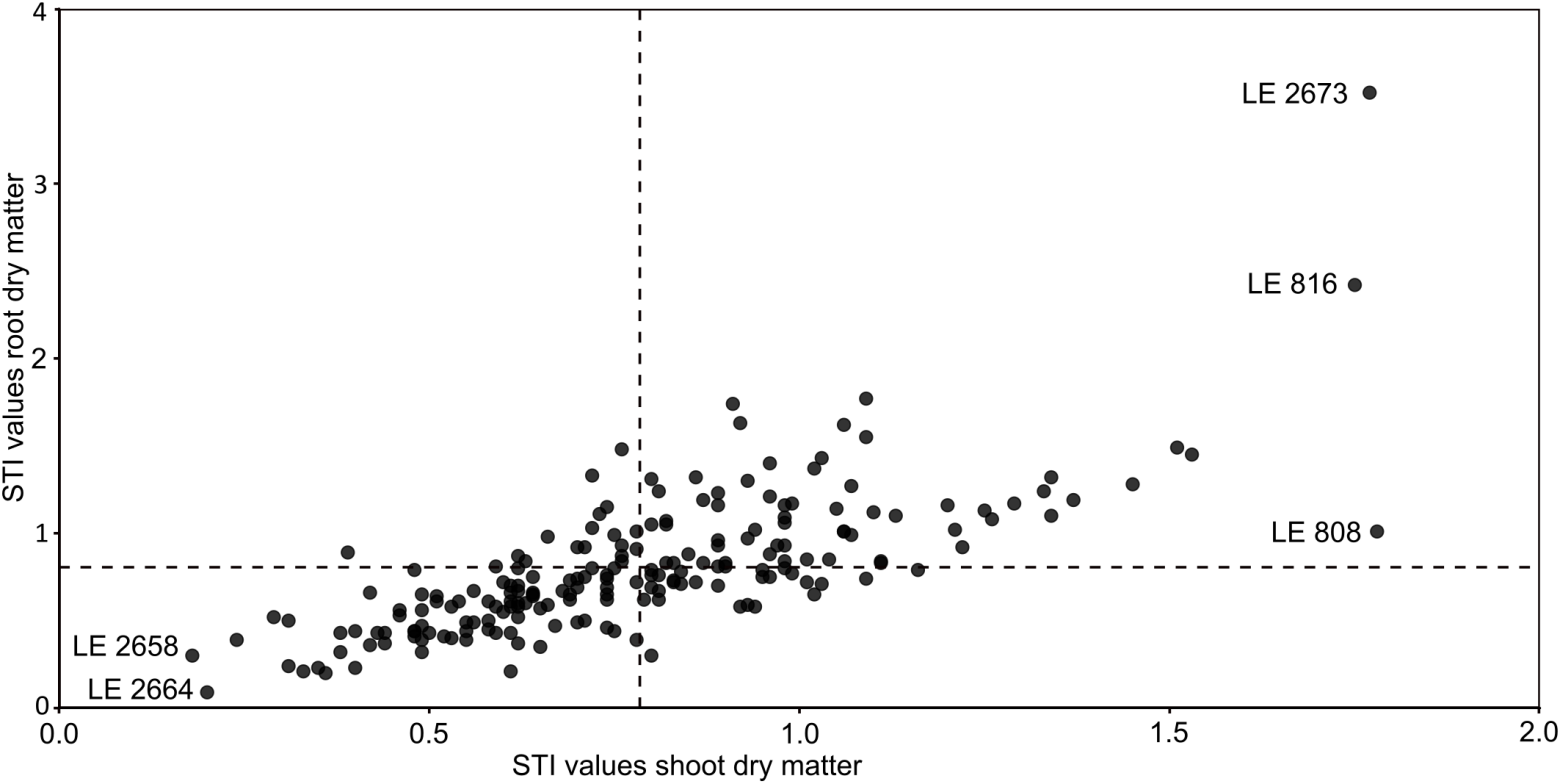
Stress tolerance index (STI) values of shoot dry matter plotted against STI values of root dry matter. Dashed lines indicate respective mean STI values.

Plants grown under HP conditions showed normal growth and healthy appearance, whereas plants under LP conditions exhibited typical P deficiency symptoms such as reduced growth. Shoot dry matter (SDM) production exhibited significant variation among accessions (p < 0.001). Under HP, SDM ranged from 0.72 to 11.60 g (average = 4.82 g), while it was lower under LP (p < 0.001), ranging from 0.50 to 8.44 g (average = 3.68 g, **Figure 1**). Root dry matter (RDM) also showed substantial variation among accessions (p < 0.001), with values under HP ranging from 0.14 to 4.49 g (average = 1.43 g) and lower values under LP from 0.07 to 3.60 g (average = 1.10 g, p < 0.001). The interaction between accession and treatment was not significant for either SDM or RDM. The root-to-shoot ratio (RSR) ranged from 0.11 to 0.52 (average = 0.30) under HP and from 0.13 to 0.68 (average = 0.30) under LP, with no significant treatment effect, whereas accession effects were significant (p < 0.001). On average, SDM and RDM decreased by approximately 24% and 23%, respectively, under LP compared with HP conditions. Although SDM and RDM generally decreased under LP conditions, a small number of accessions maintained comparable biomass under LP to that under HP (**Supplementary Table 1**). For example, accession LE 2701 showed no significant differences in shoot dry matter (LP: 5.63 g; HP: 5.50 g) or root dry matter (LP: 1.58 g; HP: 1.42 g). In contrast, accessions LE 2680 and LE 871 exhibited numerically higher root dry matter under LP (1.31 vs. 1.17 g and 1.41 vs. 1.32 g, respectively), despite reduced shoot dry matter (**Supplementary Table 1**). Stress tolerance index (STI) values calculated for SDM and RDM varied among accessions (**Figure 2**). Accessions LE 2673, LE 816, and LE 808 showed exceptional high STI values for shoot biomass, whereas LE 2658 and LE 2664 exhibited the lowest STI values. A strong positive correlation was observed between shoot and root STI values (Spearmans’s ρ = 0.77, p < 0.001).

### 3.2 Identification of candidate genes based on k-mer GWAS signals

Genome-wide association results are summarised using Manhattan plots, and corresponding quantile–quantile (QQ) plots were used to assess the distribution of observed versus expected p-values (**Supplementary Figure 3**). Genes for traits associated with the 37 significant k-mer–derived fragments were identified 50 kb upstream and downstream from the genomic coordinates of the fragments (**Supplementary Table 2**). The functional annotations of the proteins corresponding to the located genes were screened to identify candidates with biological relevance to the target traits (**Table 1**). For ΔRDM, significant k-mers were merged into one overlapping fragment mapping to the same locus on contig tig0010097 (~5600–5638). No annotated genes were identified at this locus in the current zm-4 annotation, and therefore no candidate gene was assigned. For maximum plant height under HP conditions, candidate genes were identified on chromosome 1_1 in one of the detected genomic regions, where fragments co-localized with Msa0026910, encoding a RPT2a-like 26S proteasome AAA-ATPase subunit; Msa0026930, encoding a phytochrome-interacting basic helix–loop–helix (bHLH) transcription factor; and Msa0026940, encoding a CLASP-like microtubule plus-end tracking protein (**Table 1**). For minimum plant height under LP conditions, candidate genes were identified in two regions, including a YUCCA-type flavin monooxygenase (Msa0017430) on chromosome 1_1 and a calcineurin B-like calcium sensor (Msa0424870) on chromosome 3_2. For shoot fresh matter under HP conditions, candidate genes were identified in one region on chromosome 8_1, containing three paralogous genes encoding myosin XI heavy-chain proteins (Msa1220120, Msa1220150, and Msa1220160) and a growth-regulating factor 5–like transcription factor (Msa1220170). For shoot fresh matter under LP conditions, a candidate gene encoding a GH27 α-galactosidase (Msa0074550) was identified on chromosome 1_2 within the LD window of one associated fragment. For shoot dry matter under LP conditions, one associated fragment mapped to a region on chromosome 1_1 containing an ADT1-like plastidial arogenate dehydratase (Msa0047940). In addition, for root dry matter under LP conditions, a fragment co-localized with a GT14-family Golgi glycosyltransferase (Msa0158960) on chromosome 1_4 (**Table 1**). Overall, candidate genes identified under HP conditions were mainly associated with growth regulation and cytoskeleton-related processes, whereas genes detected under LP conditions were more frequently related to hormone signalling, stress response, and metabolic adjustment pathways underlying plant responses to optimal and P limited conditions. These candidate genes were identified based on positional colocalization within LD regions and functional annotation, and therefore represent putative candidates. To confirm a direct functional role, further functional validation is required.

**Table 1:** Candidate genes located within ±50 kb LD windows around significant GWAS fragments associated with plant height and biomass traits in the *M. sativa* complex.

Trait	fragment number	-log_10_ (p-value)	Fragment genomic position	Gene ID	Gene genomic position	Functional annotation	Main biological process linked to trait	Key reference(s)
maximum plant height under HP	1	5.03	chr1_1: 51032941-51032981	Msa0026910	chr1_1: 51020065-51024894	RPT2a-like 26S proteasome AAA-ATPase subunit	meristem maintenance, organ size control via proteasome-mediated protein turnover	Ueda et al., 2011 Kurepa et al., 2008
				Msa0026930	chr1_1: 51046598-51055042	PIF1-like phytochrome-interacting bHLH TF	light/temperature-regulated elongation, GA/auxin signalling	Capella et al., 2015; Moon et al., 2008
				Msa0026940	chr1_1: 51066698-51082662	CLASP-like microtubule plus-end tracking protein	cortical microtubule organization, cell expansion, division plane orientation	Ambrose et al., 2007, 2008.
minimum plant height under LP	2	5.22	chr3_2: 75169820-75169862	Msa0424870	chr3_2: 75184493-75186197	CBL10-like calcineurin B-like Ca²^+^ sensor	Na^+^ homeostasis and salt tolerance; Ca-mediated stress signalling	Kim et al., 2007; Quan et al., 2007
	3	5.05	chr1_1: 28858224-28858255	Msa0017430	chr1_1: 28886750-28888312	YUCCA-type flavin monooxygenase	IPA-dependent auxin biosynthesis, shoot and hypocotyl elongation	Zhao et al., 2001; Müller-Moule et al., 2016
shoot fresh matter under HP	2	4.28	chr8_1: 83148789-83148841	Msa1220120	chr8_1: 83134376-83149446	Myosin XI-K-like heavy chain (Paralogous copies)	actin-based organelle transport, cell expansion, aerial organ growth	Ojangu et al., 2007, 2012; Peremyslov et al., 2008
				Msa1220150	chr8_1: 83161877-83163657			
				Msa1220160	chr8_1: 83170520-83177900			
				Msa1220170	chr8_1: 83180768-83182453	GRF5-like growth-regulating factor TF	leaf cell proliferation, chloroplast division, photosynthetic capacity	Vercruyssen et al., 2015; Beltramino et al., 2021
shoot fresh matter under LP	2	5.22	chr1_2: 49246587-49246623	Msa0074550	chr1_2: 49202329-49217071	GH27 α-galactosidase	cell-wall matrix remodeling via galactomannan/galactan turnover	Chrost et al., 2007; Zhang et al., 2021
shoot dry matter under LP	1	4.45	chr1_1: 81146399-81146433	Msa0047940	chr1_1: 81136416-81138639	ADT1-like plastidial arogenate dehydratase	phenylalanine biosynthesis feeding phenylpropanoid/lignin pathways, photosynthetic performance	Höhner et al., 2018; Chen et al., 2016
root dry matter under LP	1	5.57	chr1_4: 28947995-28948032	Msa0158960	chr1_4: 28926482-28931424	GT14-family Golgi glycosyltransferase	cell-wall polysaccharide/cellulose matrix assembly, root and culm growth	Wang et al., 2022; Zhou et al., 2009
STI for root dry matter	3	4.56	chr1_1: 6017225-6017259	Msa0003450	chr1_1: 5968700-5973519	JMT-like jasmonic acid carboxyl methyltransferase	JA to MeJA conversion, biomass allocation and carbon partitioning	Seo et al., 2001; Qi et al., 2016
	5	4.17	chr1_1: 46131987-46132018	Msa0024030	chr1_1: 46120252-46124953	LOG-type cytokinin riboside 5’-monophosphate phosphoribohydrolase	cytokinin activation, root and shoot meristem activity	Kuroha et al., 2009; Tokunaga et al., 2011

### 3.3 Shoot P content and P utilisation efficiency

Shoot P content and PUE were evaluated in a subset of each 10 accessions highly responsive to LP and stable across both P conditions, representing diverse biological statuses and geographic origins. The selection of this subset was based on ΔRDM, which distinguishes accessions with contrasting responses to P availability better than STI, which includes overall productivity.

**Figure 3:**
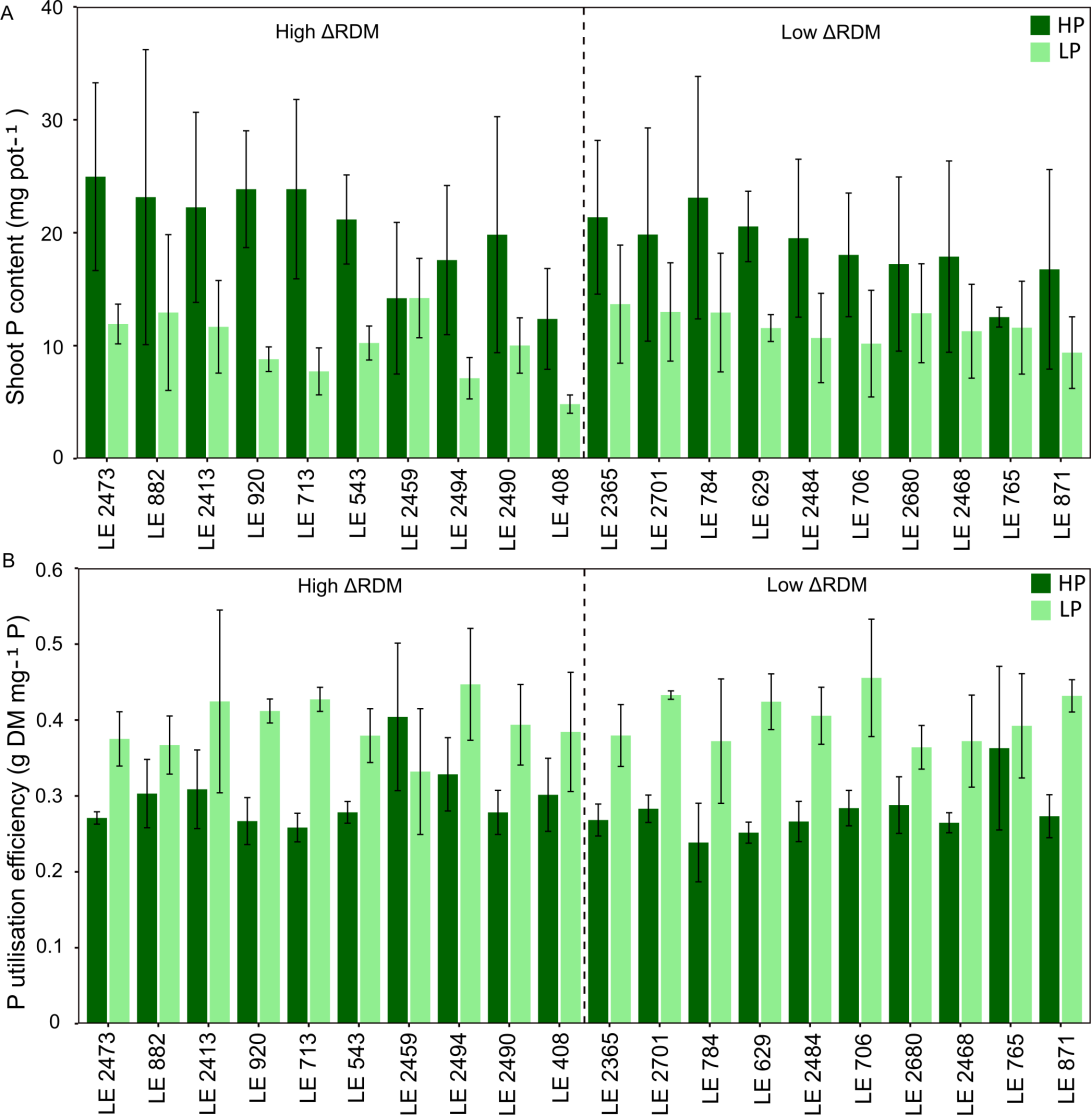
**(A)** Total shoot phosphorus (P) content (mg per pot) and **(B)** P utilisation efficiency (g shoot dry matter per mg P in shoot) under high P and low P conditions. Bars represent mean values ± standard deviation (n=3). Accessions are ordered according to their root dry matter response to P supply, with the first ten accessions representing the ‘High ΔRDM’ group and the subsequent ten accessions representing the ‘Low ΔRDM’ group.

Under LP conditions, accessions classified as “High ΔRDM” exhibited biomass reductions, with average decreases in RDM and SDM of 41.4% and 36.0% compared with HP, respectively. In contrast, “Low ΔRDM” accessions showed comparatively minor biomass changes, with average RDM and SDM reductions of −2.3% and 6.1%, respectively (**Supplementary Table 1**). Shoot P content differed strongly between P treatments (p < 0.001), with consistently lower values observed under LP than under HP conditions (**Figure 3A**). Within the “High ΔRDM” group, shoot P content under HP conditions ranged from 6.78 to 36.5 mg pot^-^¹ (average = 20.3 mg pot^-^¹), while under LP it ranged from 4.20 to 20.9 mg pot^-^¹ (average = 9.94 mg pot^-^¹). This corresponded to an average reduction of 51.1%. A similar pattern was evident in the “Low ΔRDM” group, where shoot P content ranged from 7.91 to 34.7 mg pot^-^¹ (average = 18.7 mg pot^-^¹) under HP and from 4.80 to 19.7 mg pot^-^¹ (average = 11.7 mg pot^-^¹) under LP, resulting in an average reduction of 37.3%. In addition to the treatment effect, both ΔRDM group (p = 0.034) and the group × treatment interaction (p = 0.034) were significant. However, when treatments were evaluated separately, “High and Low ΔRDM” groups did not differ significantly under either HP or LP conditions (p > 0.10). By comparison, PUE exhibited a consistent response across groups (**Figure 3B**). Across all accessions, PUE was significantly higher under LP than under HP conditions (p < 0.001). In the “High ΔRDM” group, PUE ranged from 0.23 to 0.48 g DM mg^-^¹ P under HP (average = 0.30 g DM mg^-^¹ P) and from 0.26 to 0.56 g DM mg^-^¹ P under LP (average = 0.40 g DM mg^-^¹ P). Likewise, in the “Low ΔRDM” group, PUE ranged from 0.20 to 0.48 g DM mg^-^¹ P under HP (average = 0.28 g DM mg^-^¹ P) and from 0.30 to 0.53 g DM mg^-^¹ P under LP (average = 0.40 g DM mg^-^¹ P). Neither ΔRDM group (p = 0.35) nor the group × treatment interaction (p = 0.13) was significant for PUE. Correlation analysis based on HP–LP differences further supported this distinction between traits. Reductions in shoot P content were strongly associated with differences in SDM and RDM (Spearman’s ρ = 0.80 and 0.64, respectively; p < 0.01), whereas changes in PUE were not significantly correlated with changes in biomass or shoot P content (p > 0.10, **Supplementary Figure 2**).

### 3.4 Plant performance under P deficiency in the rhizotron experiment

**Figure 4:**
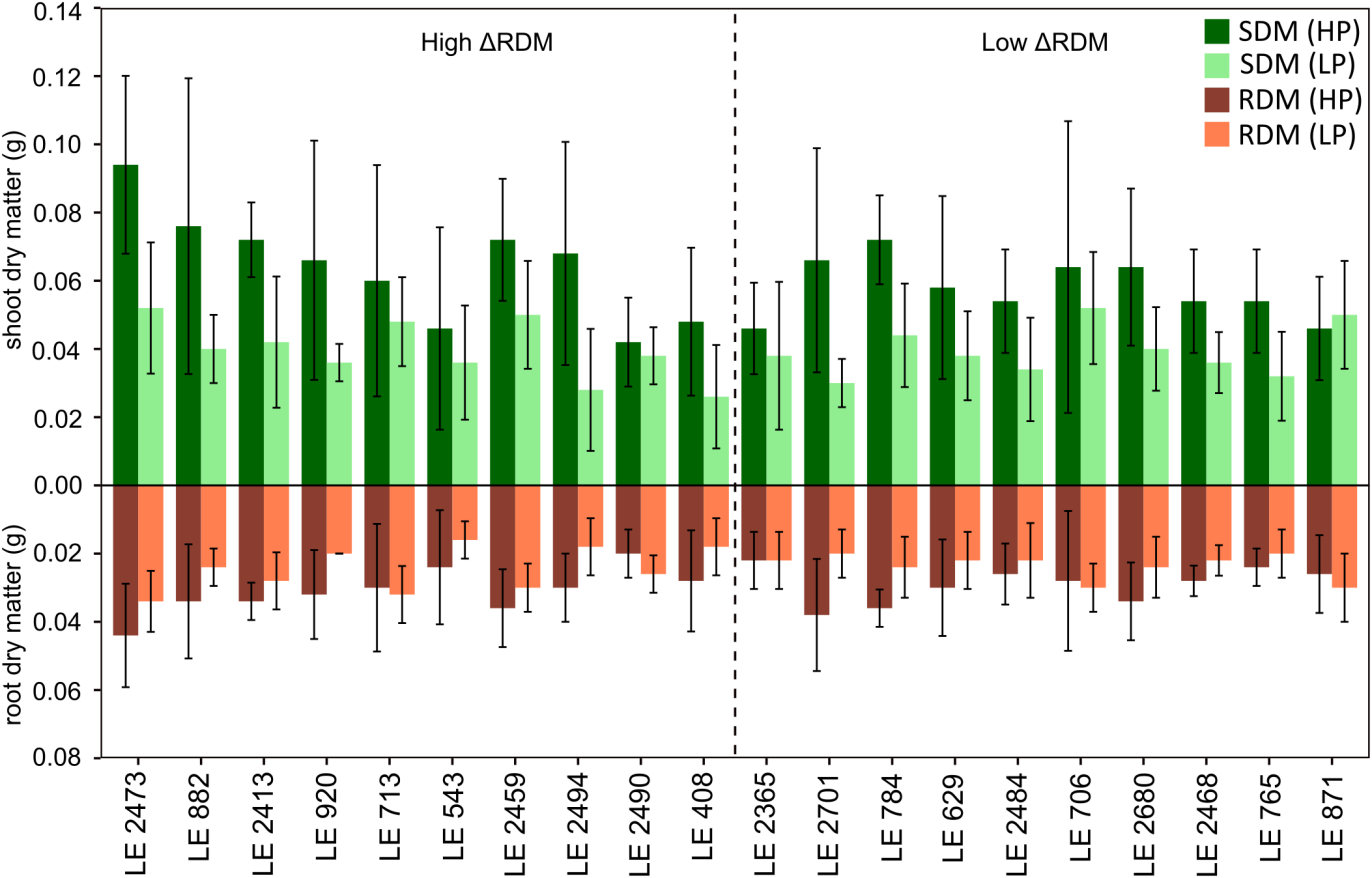
Bar plot showing shoot and root dry matter production under high phosphorus (HP) and low phosphorus (LP) conditions for 20 selected accessions in rhizotrons. Bars indicate mean values ± standard deviation (n = 5). Accessions are ordered according to their root dry matter response to P supply in the pot experiment, with a dashed line separating “High and Low ΔRDM” groups. .

To complement the pot experiment, SDM, RDM and RSA were evaluated in the same subset of 20 accessions using a rhizotron-based system (**Figure 4**). In the ‘High ΔRDM’ group, SDM under HP conditions ranged from 0.04 to 0.09 g (average = 0.06 g), whereas under LP it ranged from 0.03 to 0.05 g (average = 0.04 g), corresponding to an average reduction of 36.2%. Root dry matter ranged from 0.02 to 0.04 g under HP (average = 0.03 g) and from 0.02 to 0.03 g under LP (average = 0.02 g), representing an average reduction of 22.3%. Consistent with the stronger reduction in SDM relative to RDM, RSR increased under LP, rising from an average of 0.50 under HP to 0.62 under LP (+23.6%). In the “Low ΔRDM” group, SDM ranged from 0.04 to 0.07 g under HP (average = 0.06 g) and from 0.03 to 0.05 g under LP (average = 0.04 g), corresponding to an average reduction of 30.9%. Root dry matter ranged from 0.02 to 0.04 g under HP (average = 0.03 g) and from 0.02 to 0.03 g under LP (average = 0.02 g), resulting in an average reduction of 18.3%. Similar to the “High ΔRDM” group, RSR increased under LP from an average of 0.50 to 0.61 (+22.9%). ANOVA revealed a strong effect of treatment on SDM, RDM, and RSR (**Figure 4**; p < 0.001 for all traits). ΔRDM group effect was significant for SDM and RDM (p < 0.01), but not for RSR. No significant treatment × group interactions were detected for any trait. Significant accession effects were observed for SDM and RDM (p < 0.01), whereas accession effects were not significant for RSR.

Root system architectural (RSA) traits responded differentially to P supply (**Table 2**). Traits related to overall root system size, including total root length, volume, surface area, area, and diameter, showed significant treatment effects with higher values under HP than under LP conditions. Specifically, root length decreased from 1768 mm under HP to 1621 mm under LP (p = 0.023), root volume from 355 to 301 mm³ (p = 0.002), surface area from 2785 to 2461 mm² (p = 0.005), and root diameter from 0.51 to 0.48 mm (p < 0.001). In contrast, traits describing spatial root deployment exhibited weaker or contrasting responses to P supply. Rooting width and convex hull width showed small but significant increase under LP compared to HP (rooting width: 121 vs. 116 mm, p = 0.040; convex hull width: 125 vs. 119 mm, p = 0.020), whereas rooting depth and convex hull height did not differ significantly between treatments (p = 0.38). Convex hull area was likewise unaffected by P availability (p = 0.64). Efficiency-related RSA traits showed the opposite trend to size-related traits. Specific root length increased from 5.33 mm mm^-^³ under HP to 5.68 mm mm^-^³ under LP (p = 0.015), and specific convex hull area, the ratio of convex hull area to root area, increased from 25.6 to 30.6 mm² mm^-^² (p < 0.001), indicating a shift toward finer and more spatially efficient root systems under P limitation.

**Table 2:** Summary of root system architecture (RSA traits) measured in rhizotrons for 20 accessions grown under high phosphorus (HP) and low phosphorus (LP) conditions.

Trait	Treatment	Min	Max	Mean	P value (T)	P value (A)	P value (T × A)
length (mm)	HP	622	3399	1768	*	ns	ns
	LP	601	2775	1621			
volume (mm^3^)	HP	85.6	962	355	**	ns	ns
	LP	90.9	603	301			
diameter (mm)	HP	0.38	0.67	0.51	***	ns	ns
	LP	0.39	0.60	0.48			
rooting width (mm)	HP	64.4	159	116	*	*	ns
	LP	63.5	148	121			
rooting depth (mm)	HP	205	401	339	ns	ns	ns
	LP	177	401	334			
convex hull width (mm)	HP	63.7	174	119	*	**	ns
	LP	63.5	154	125			
convex hull height (mm)	HP	207	408	344	ns	ns	ns
	LP	184	408	342			
surface area (mm^2^)	HP	1019	5923	2785	**	ns	ns
	LP	821	4222	2461			
area (mm^2^)	HP	361	2022	989	**	ns	ns
	LP	288	1506	879			
convex hull area (mm^2^)	HP	10270	40597	25360	ns	**	ns
	LP	9372	40658	25765			
specific root length (mm mm^-3^)	HP	2.86	11.3	5.33	*	ns	ns
	LP	3.56	8.36	5.68			
specific convex hull area (mm^2^ mm^-2^)	HP	15.4	46.7	26.9	***	**	ns
	LP	18.6	47.6	30.6			

Values indicate minimum (min), maximum (max), mean per trait and treatment (five replicates from 20 accessions). p-values refer to treatment (T), accession (A), and their interaction (T × A). Significance codes: ns, not significant (p ≥ 0.05); * p < 0.05; ** p < 0.01; *** p < 0.001.

**Figure 5:**
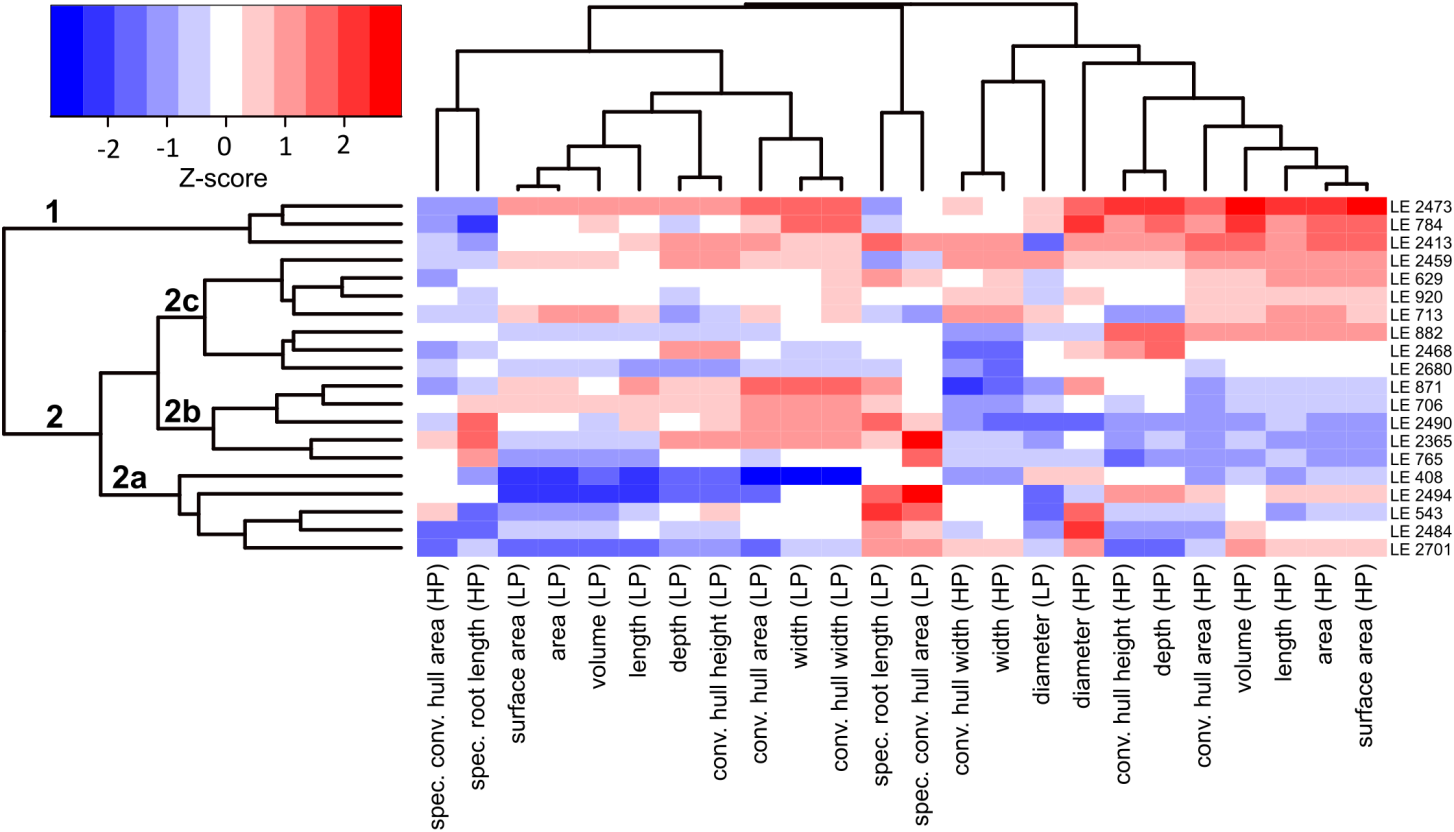
Heatmap of root system architectural (RSA) traits across 20 *Medicago* accessions under high phosphorus (HP) and low phosphorus (LP) conditions. Colors represent Z-scores (red = above mean, blue = below mean) of each trait, enabling comparison across accessions and treatments. Traits and accessions are clustered hierarchical based on Euclidean distances.

Hierarchical clustering of standardized RSA traits across HP and LP conditions separated the accessions into two main clusters (**Figure 5**). Cluster 1 comprised three accessions, LE 2473 and LE 2413 from the “High ΔRDM” group, and LE 784 from the “Low ΔRDM” group, and was characterised by consistently higher values for traits describing overall root system size and spatial extent, particularly under HP conditions. These accessions showed elevated total root length, root surface area, root volume, convex hull area, rooting depth, and root diameter compared with the remaining accessions an a reduced but still large root system under LP. Cluster 2 included the remaining accessions from both ΔRDM groups and was characterised by comparatively smaller root systems. Within sub-cluster 2a, accessions LE 408, LE 2494, and LE 543 (“High ΔRDM” group) and LE 2484 and LE 2701 (“Low ΔRDM” group) displayed particularly low values for root system size-related traits, while exhibiting relatively higher values for efficiency related traits such as specific root length and specific convex hull area, especially under LP conditions, and a further reduction of root system size under LP. The subset within sub-cluster 2b, LE 871, LE 706, LE 2365, and LE 765 (“Low ΔRDM” group) together with LE 2490 (“High ΔRDM” group), showed equal or higher values for selected root system size traits under LP compared with HP. The accessions in sub-cluster 2c, LE 2459, LE 920, LE 713, and LE 882 (“High ΔRDM” group) and LE 629, LE 2468, and LE 2680 (“Low ΔRDM” group), displayed intermediate RSA trait values without a consistent directional shift between P treatments.

## 4 Discussion

### 4.1 Shoot growth responses to low P conditions

This study demonstrates considerable intraspecific variability among diverse accessions of the *Medicago sativa* complex in their growth responses to P deprivation. The overall reduction in SDM, RDM, and plant height observed across the panel represents a typical plant response to low P availability and has previously been reported in alfalfa (Pang et al., 2010; He et al., 2017). Shoot P content declined markedly under low P conditions (**Figure 3A**), reflecting reduced external P availability and uptake which is a response commonly observed in legumes experiencing P deficiency (Jeong et al., 2017). In contrast, PUE increased significantly under low P (**Figure 3B**), indicating that plants partially compensated for reduced P supply through improved internal remobilisation of available P, reduced tissue P demand, and preferential allocation of P to metabolically active tissues. These mechanisms have been proposed as central components of P efficiency in legumes (Jeong et al., 2017; Paul et al., 2023) and were consistent over the two groups with high and low biomass responsiveness to LP. Thus, shoot P content followed largely shoot biomass within the treatments. Accession-specific growth responses not only enabled the identification of contrasting groups for detailed analyses and promising candidates for breeding purposes (see below), but also facilitated the selection of candidate genes associated with the studied traits through k-mer–based GWAS. Because our study focuses on growth-related traits rather than directly on P content or uptake, the identified genomic regions likely reflect regulatory variation influencing growth expression and phenotypic plasticity across contrasting nutrient environments. The observed associations suggest that the maintenance of growth under P limitation is achieved primarily through coordinated hormonal regulation and structural adjustment, rather than solely through enhanced P uptake. This shows the fundamental constraints on P acquisition in soil, where P is poorly mobile and its transfer to roots depends largely on diffusion processes in the rhizosphere (Lambers et al., 2006; Hinsinger et al., 2011). Under such conditions, increasing uptake capacity alone may not be sufficient, and plants rely on additional adaptive strategies such as optimising internal P use efficiency, adjusting growth and resource allocation patterns (Lynch, 2019). Under HP conditions, k-mer fragments associated with maximum plant height mapped to genomic regions containing candidate genes involved in proteasome-mediated protein turnover (Ueda et al., 2011; Kurepa et al., 2008), light-regulated elongation signaling (Moon et al., 2008; Capella et al., 2015), and microtubule organisation (Ambrose et al., 2007, 2008). These functional categories are directly linked to meristem activity and directional cell expansion, processes that likely underpin the large range (**Figure 1**) in SDM and RDM observed under optimal P supply. In contrast, k-mer fragments associated with plant height and root biomass under LP mapped to genomic regions containing candidate genes involved in hormonal regulation and cell-wall modification, including auxin biosynthesis (Zhao et al., 2001), cytokinin activation (Tokunaga et al., 2011), jasmonate metabolism (Seo et al., 2001; Qi et al., 2016), calcium-mediated signalling (Kim et al., 2007; Quan et al., 2007), and glycosyltransferase-mediated cell-wall assembly (Zhou et al., 2009; Wang et al., 2022). These functional categories are closely associated with root–shoot balance, stress-responsive growth adjustment, and structural reinforcement, which may contribute to the partial maintenance of biomass observed in several accessions under LP despite the average 24% and 23% reductions in SDM and RDM, respectively. Beyond plant height, genomic regions associated with shoot biomass highlighted regulators of photosynthetic performance and cellular expansion. Plastidial arogenate dehydratases (Chen et al., 2016; Höhner et al., 2018), GRF5-like transcription factors (Vercruyssen et al., 2015; Beltramino et al., 2021), myosin XI motors (Ojangu et al., 2007; Peremyslov et al., 2008; Ojangu et al., 2012), and GH27 α-galactosidases (Chrost et al., 2007; Zhang et al., 2021) are all associated with sustained cell expansion and biomass accumulation. While these loci represent biologically plausible candidates, their identification is based on positional colocalization within LD intervals and homology-based functional annotation, which does not establish the accurate biological role. Therefore, functional validation in alfalfa will be required to confirm their direct contribution to P-responsive growth, especially since all accessions are populations, i.e. mixtures of genotypes, and traits measurements as well as DNA sampling was not done on single plants.

### 4.2 Root growth and biomass allocation as functional drivers of shoot performance under P deficiency

Root traits play a central role in shaping shoot performance under P limitation by constraining P acquisition and mediating biomass allocation. In the present study, reductions in RDM under low P occurred in parallel with declines in shoot biomass, indicating coordinated suppression of above- and below-ground growth under P stress, a response widely reported in alfalfa and other legume species (Fan et al., 2015; He et al., 2017; Zhang et al., 2024; Fan et al., 2025). However, RDM responses to low P varied markedly among accessions, ranging from strong reductions to slight increases. A comparison of selected contrasting accessions showed that differences in RDM under LP were closely mirrored by changes in SDM and shoot P content. Correlation analysis (**Supplementary Figure 2**) based on HP–LP differences confirmed this pattern, with strong positive associations between RDM and SDM and between biomass traits and shoot P, indicating coordinated above- and below-ground growth responses linked to P acquisition. In contrast, changes in PUE were not significantly correlated with SDM, RDM, or shoot P, suggesting that internal P use efficiency operates largely independently of biomass reduction and shoot P uptake under P stress. This independence suggests that PUE may reflect metabolic adjustments downstream of P acquisition, including internal P remobilisation and optimisation of cellular P utilisation. Mechanisms such as enhanced recycling of inorganic phosphate and reduced P demand have been proposed as central components of internal P efficiency in plants under nutrient limitation (Veneklaas et al., 2012; Lambers et al., 2015). The clear separation between biomass-associated P uptake responses and PUE observed in this study is consistent with previous work highlighting the conceptual and methodological distinction between PAE and internal PUE, which are often confounded in soil-based systems (Rose et al., 2011).

Results from the rhizotron experiment complemented the pot experiment by confirming clear treatment effects on SDM and RDM production (**Figure 4**). However, accession-specific differentiation in biomass was attenuated in the rhizotron system. This discrepancy between experimental systems may partly reflect differences in root growth space and substrate conditions. In pots, roots explore a larger three-dimensional soil volume, whereas rhizotrons constrain root development within a narrow two-dimensional space. Such differences can influence root architecture, soil exploration patterns, and P diffusion around roots, potentially reducing the expression of accession-dependent differences in P acquisition (Watt et al., 2013). In addition to these structural constraints, differences in P supply between the systems may also have contributed to the observed patterns. Specifically, the higher effective P supply under LP in the rhizotron system compared with the pot experiment may have contributed to the reduced differentiation among accessions. Consistent with this, the pot experiment showed strongly divergent RDM responses under LP between the “High and Low ΔRDM” groups, whereas the range of HP–LP contrasts was smaller in the rhizotrons, resulting in reduced separation between the two groups. Such system-dependent expression of genetic variation has previously been reported for root-related traits under controlled growth conditions (Ticconi and Abel, 2004). Plants in the rhizotrons were younger and less developed than those in the pots, and differences in developmental stage have been shown to influence the expression of root traits and genotype effects across experimental systems (Odone and Thorup-Kristensen, 2025; Watt et al., 2013). Moreover, nutrient demand increases with plant growth and biomass accumulation, potentially intensifying nutrient limitation at later developmental stages. Accordingly, RDM responses in the rhizotron system did not fully reproduce the ΔRDM-based groupings identified in the pot experiment: both “High and Low ΔRDM” groups exhibited reductions in RDM under LP, accompanied by a consistent increase in RSR. This proportional shift in carbon allocation toward roots represents a compensatory adjustment to nutrient limitation, enabling maintenance of soil exploration capacity despite declining total biomass and improving the ability of roots to access immobile nutrients such as P (Mollier and Pellerin, 1999; Lynch and Brown, 2001; Ticconi and Abel, 2004; Vengavasi and Pandey, 2016).

Analysis of RSA traits revealed additional adaptive patterns. Size-related parameters, including total root length, surface area, volume, and mean diameter, declined under LP, whereas efficiency-related traits showed the opposite trend. In particular, increases in specific root length and specific convex hull area (**Table 2**) indicate a shift toward finer and more spatially efficient root systems, enhancing soil exploration per unit carbon investment. This adjustment reflects a common adaptive strategy among plants growing in nutrient-poor environments, where increasing root surface area relative to biomass improves nutrient foraging efficiency without large increases in carbon expenditure (Lynch, 2019). Additional root distribution patterns further supported this adjustment strategy: horizontal expansion increased under LP, while rooting depth remained unchanged, leading to enhanced foraging of upper soil layers. Because P is an immobile nutrient enriched in topsoil horizons (Hinsinger et al., 2011), and was applied at 2 cm depth in our system, a shallower, laterally expanding root system constitutes a functionally advantageous response under LP conditions (Lynch, 2019). Importantly, accession responses were not uniform. Four broad RSA response types could be distinguished: two groups with different initial root system sizes that exhibited absolute reductions under LP, one group showing an absolute increase in root system size, and one group with no consistent response pattern. The distinct RSA response types identified in this study align with previous observations of genotype-dependent root architectural plasticity under P limitation in sorghum (Parra-Londono et al., 2018), potato (Kirchgesser et al., 2023), and alfalfa (Bucciarelli et al., 2021; Pan et al., 2023). Nevertheless, morphological adjustments such as increased root fineness or altered spatial distribution may involve trade-offs with physiological efficiency mechanisms (Wen et al., 2019), which were not assessed in the present study.

### 4.3 Intraspecific variability

Our study revealed substantial variability in growth responses to contrasting P supply within the *Medicago sativa* complex at the early developmental stage. For the development of P efficient cultivars, both high overall productivity and stability across P environments are essential. These aspects are integrated in the stress tolerance index (STI), where higher values indicate a combination of high yield potential and stability under stress (Bhandari et al., 2024). As alfalfa is harvested for its aboveground biomass, SDM-derived STI directly reflects agronomic performance. Accordingly, LE 2673, LE 816, and LE 808 were identified as promising candidates (**Figure 2**). LE 2673 represents wild material collected in Tunisia, LE 816 corresponds to the cultivar “Slovenska Podunajska,” and LE 808 is the breeding hybrid “Severnaja Gibridnaja 69” from the former Czechoslovakia and Soviet Union, respectively. The superior performance of these accessions across contrasting P conditions demonstrates that favorable P efficiency can arise from diverse genetic backgrounds and biological statuses rather than from a single domestication or breeding trajectory. Similar patterns have been reported in other forage and grain legumes, where both wild and cultivated germplasm contributed useful alleles for nutrient efficiency (Pang et al., 2018).

In contrast, very low STI values were observed for LE 2658 and LE 2664, both belonging to *Medicago sativa* ssp. *caerulea* from Tajikistan and Kazakhstan, respectively. As the cultivated tetraploid *M. sativa* ssp. *sativa* originated from the diploid ssp. *caerulea* (Yu et al., 2017), and polyploidization in *Medicago* has been associated with increased vigor, larger organ size and biomass production compared with diploid progenitors (Innes et al., 2020), the comparatively low productivity of most ssp. *caerulea* accessions in this study is consistent with ploidy-related differences in growth potential. Nevertheless, such material may harbour specific adaptive traits that are valuable for introgression into high-yielding genetic backgrounds.

A small subset of accessions maintained comparable aboveground biomass under LP and HP in the pot experiment, predominantly European cultivars. Many of these low-responsive accessions also exhibited limited belowground plasticity and were therefore selected for detailed root system analyses (e.g., LE 2701, LE 765, LE 2680, and LE 706). While their STI values were generally moderate, LE 2701 combined relatively stable biomass production with above-average STI, indicating potential as a stable, high-yielding breeding resource. However, this apparent stability was not consistently reproduced in the rhizotron experiment, underlining the system-dependent expression of P response traits and the need for multi-environment validation.

Taken together, these results indicate that P efficiency in alfalfa is not governed by a single optimal phenotype but can emerge from alternative trait combinations balancing carbon allocation, root architectural plasticity, and internal P utilisation processes. This aligns with recent findings in forage legumes demonstrating multiple adaptive pathways to nutrient stress tolerance (Pan et al., 2023). Further physiological analyses will be necessary to disentangle acquisition- versus utilisation-driven mechanisms. Moreover, because early-stage pot and rhizotron experiments may not fully capture field-level responses, particularly for accessions not adapted to local climatic conditions (Hu et al., 2023), validation under field conditions and at later developmental stages is essential before integrating selected accessions into breeding programs.

Despite these insights, limitations of the present study should be acknowledged. Phenotypic measurements were conducted at early developmental stages under controlled conditions, which may not fully reflect long-term plant performance under field environments. However, the combination of pot and complementary rhizotron experimental systems enabled a precise and reproducible assessment of accession-specific responses to P availability, with reduced environmental variation across experiments. In addition, alfalfa accessions represent genetically heterogeneous populations typical of this outcrossing autotetraploid species, meaning that intra-accession genetic variation may contribute to the observed phenotypic responses. While this increases phenotypic variability, the use of a diverse panel and consistent phenotyping across experiments allowed the identification of stable response patterns. Moreover, the GBS data were generated from pooled samples, capturing the overall allelic composition of each accession. This heterogeneity reflects the genetic complexity relevant for breeding applications. The candidate genes identified through k-mer GWAS represent genomic associations and require functional validation before their biological roles in P responses can be confirmed. The 100 kb window (± 50 kb) used to find candidate genes focuses on the most likely genes, but it may not capture relevant genes located further away from the associated loci if the actual LD block is larger than 100kb. Nevertheless, our findings provide an integrated framework for understanding P efficiency in *M. sativa* germplasm and offer a trait-based basis for breeding approaches aimed at improving forage performance under P limited conditions. Future studies combining field validation, detailed physiological measurements, and functional genomic analyses will be necessary to confirm the mechanisms underlying P efficiency and to translate these findings into breeding strategies for improved P efficiency in alfalfa.

## Data Availability

The original contributions presented in the study are included in the article/**Supplementary Material**. Further inquiries can be directed to the corresponding author.
