# Supplementary material for: Effect of phosphorus deficiency on biomass and root system architecture in diverse Medicago accessions
Source: Front Plant Sci. 2026 Apr 23;17:1812278. doi: 10.3389/fpls.2026.1812278 (PMC13149422; doi:10.3389/fpls.2026.1812278)
Supplement: Supplementary file 1 [file DataSheet1.pdf]

Supplementary Figures

Effect of phosphorus deficiency on biomass and root system architecture in diverse *Medicago* accessions

Nagarjun Devabhakthini, Bettina Eichler-Löbermann, Reza Haghi, Evelin Willner, Klaus J. Dehmer, Mareike Kavka

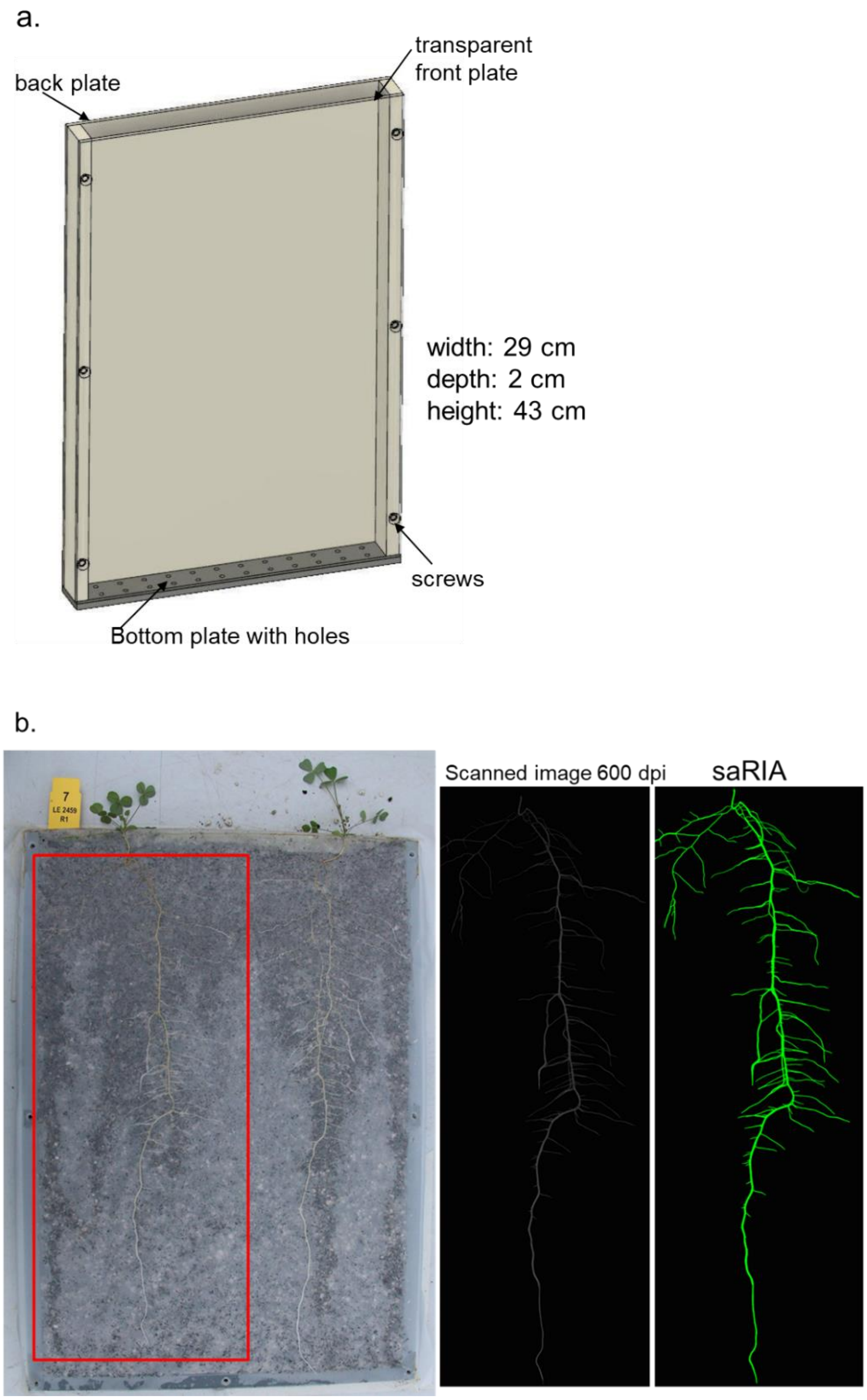

Figure S1: a) Schematic representation of the rhizotron used for root phenotyping, showing the transparent front panel, back plate, side screws, and perforated bottom plate for drainage. b) Example of a rhizotron-grown plant showing the visible root system on the nylon sheet (left), followed by scanned root image and the same image used for root system architecture analysis using saRIA (right).

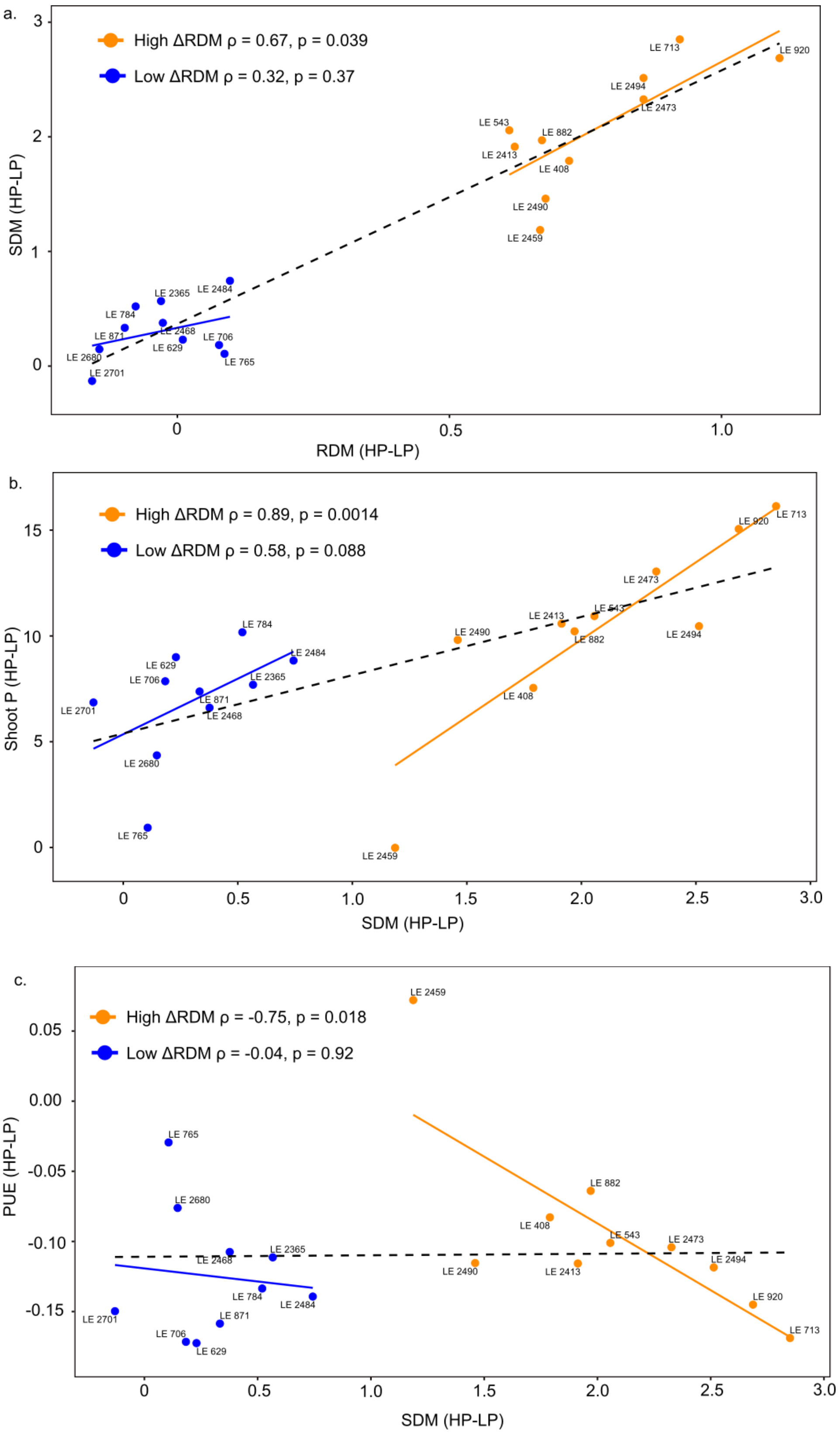

Figure S2: Spearman correlation analyses of trait responses (HP–LP differences) (a) RDM vs. SDM, (b) SDM vs. shoot P content, (c) SDM vs. PUE within “High ΔRDM” (dark orange) and “Low ΔRDM” (blue) accession groups. Each point represents one accession (mean value of three replicates), and solid lines indicate group-specific linear regression trends.

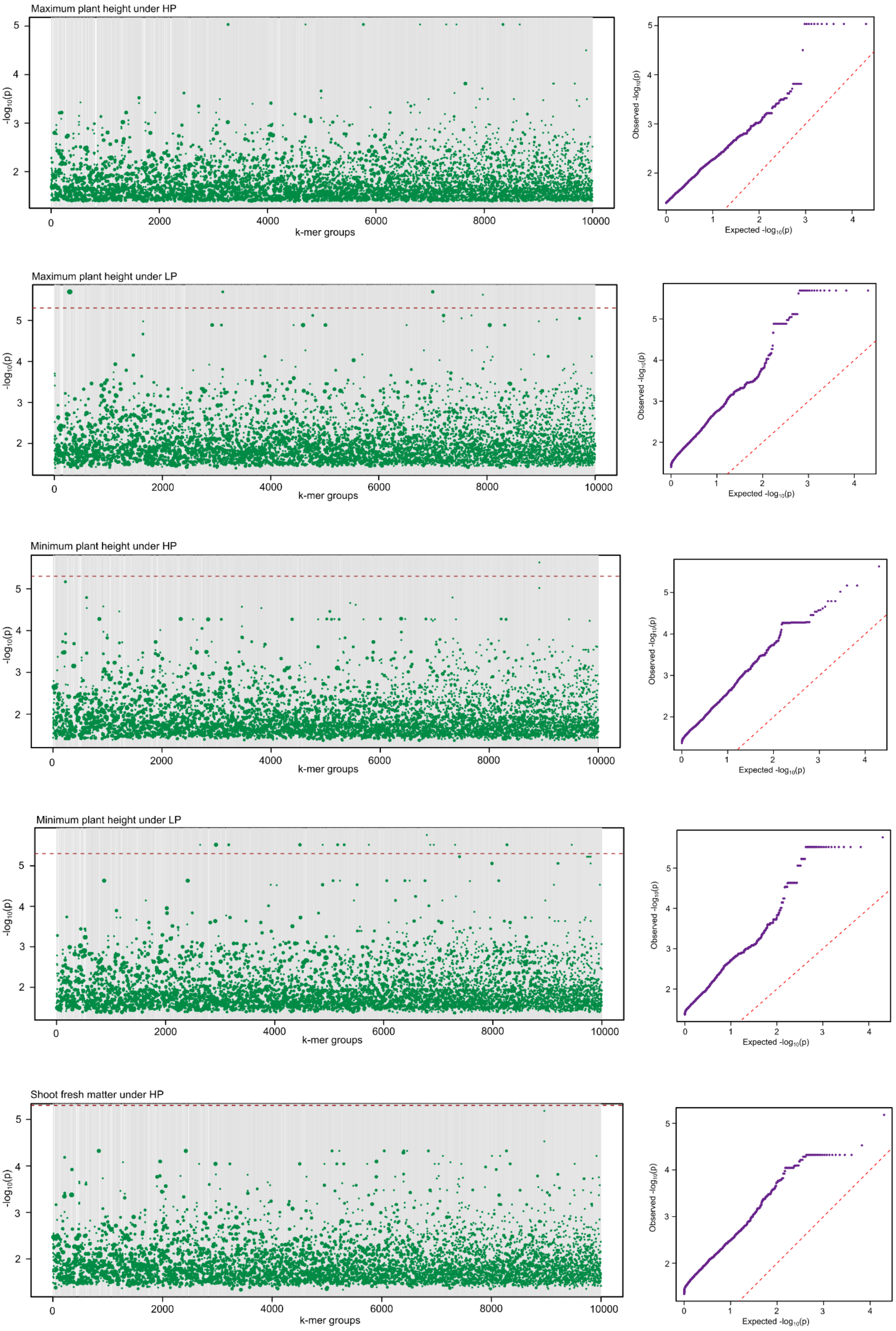
Maximum plant height under HP
Maximum plant height under LP
Minimum plant height under HP
Minimum plant height under LP
Shoot fresh matter under HP
-log10(p)
k-mer groups
Observed -log10(p)
Expected -log10(p)

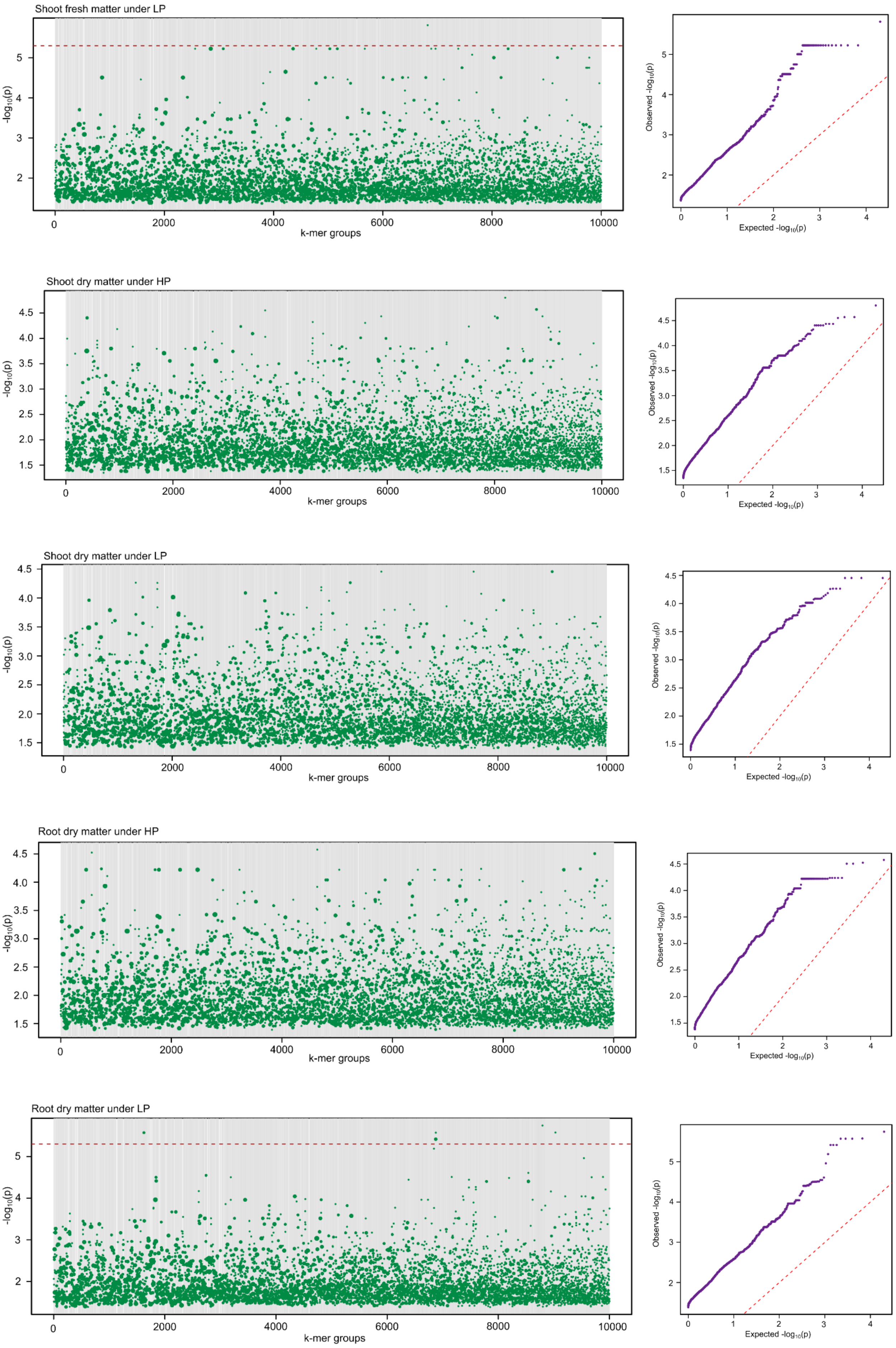

Shoot fresh matter under LP
Shoot dry matter under HP
Shoot dry matter under LP
Root dry matter under HP
Root dry matter under LP
-log10(p)
k-mer groups
0
2000
4000
6000
8000
10000
Observed -log10(p)
Expected -log10(p)

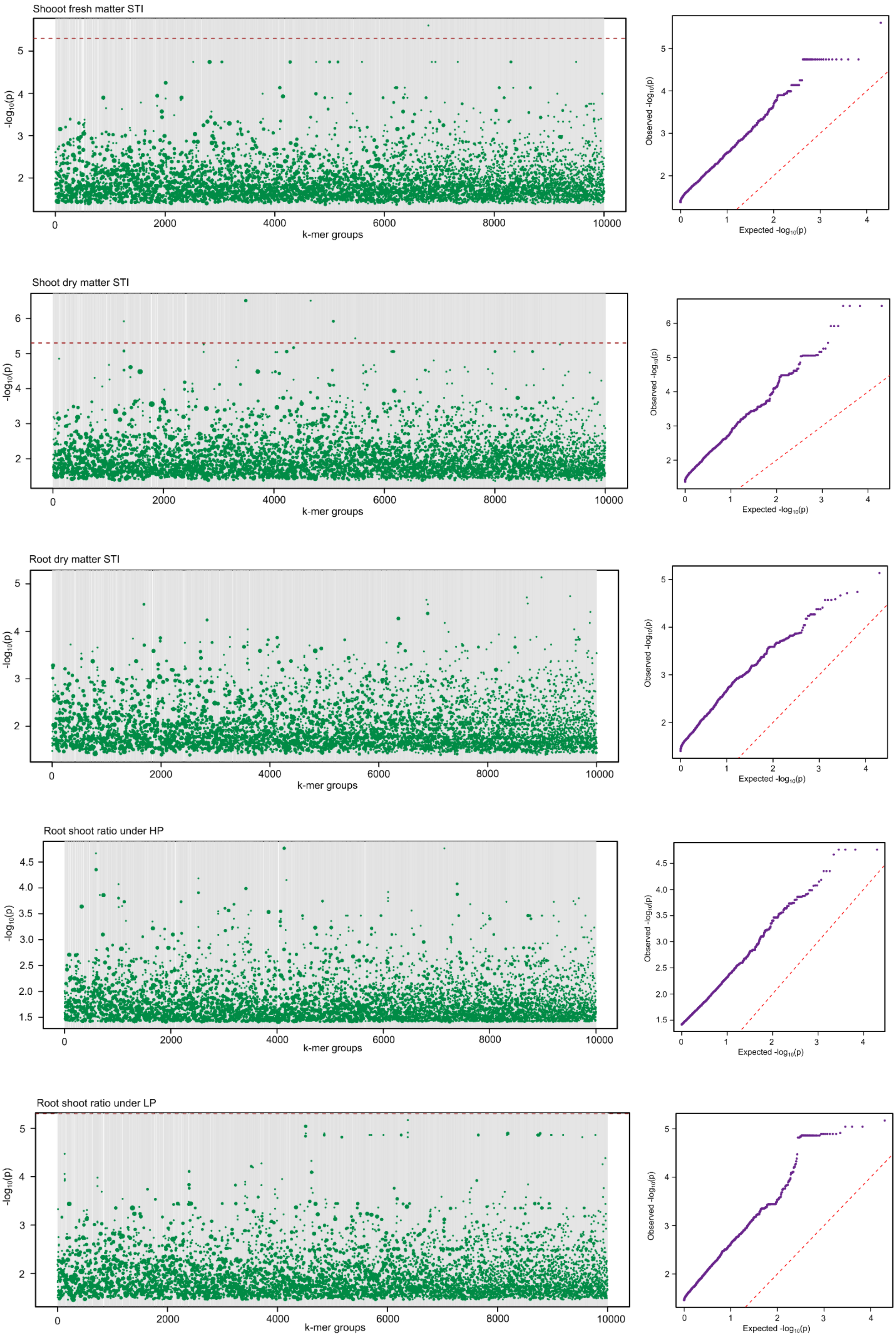

Shooot fresh matter STI
Shoot dry matter STI
Root dry matter STI
Root shoot ratio under HP
Root shoot ratio under LP
-log10(p)
k-mer groups
Observed -log10(p)
Expected -log10(p)

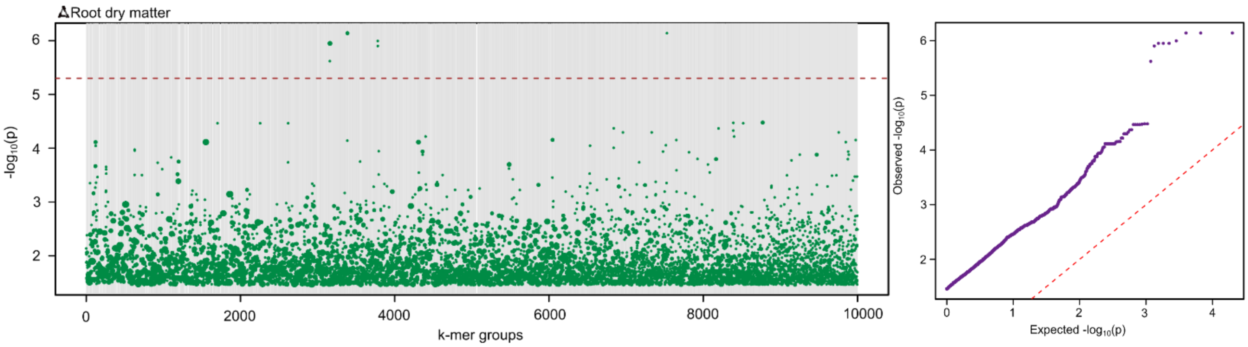

Figure S3: Manhattan and quantile–quantile (QQ) plots of k-mer-based genome-wide association analyses for selected traits under contrasting P conditions. In the Manhattan plots, each point represents an individual k-mer, plotted according to its association significance $-\log_{10}$(p-values). The x-axis represents k-mer groups, and the y-axis shows $-\log_{10}$(p-values). The red dashed line indicates the Bonferroni significance threshold ($p = 5 \times 10^{-6}$). The size of the green dots reflects the number of k-mers with the same p-value. QQ plots compare the observed versus expected $-\log_{10}$(p-values) to assess deviations from the null distribution, with upward deviations indicating potential true associations.
